# Combined Red Clover isoflavones and probiotics potently reduce menopausal vasomotor symptoms

**DOI:** 10.1371/journal.pone.0176590

**Published:** 2017-06-07

**Authors:** Max Norman Tandrup Lambert, Anne Cathrine Thorup, Esben Søvsø Szoscka Hansen, Per Bendix Jeppesen

**Affiliations:** 1 Department of Endocrinology and Internal Medicine, Aarhus University Hospital, Aarhus, Denmark; 2 MR Research Centre, Aarhus University Hospital, Skejby, Denmark; Colorado State University, UNITED STATES

## Abstract

**Background:**

Natural estrogen decline leads to vasomotor symptoms (VMS). Hormone therapy alleviates symptoms but increases cancer risk. Effective treatments against VMS with minimal cancer risks are needed. We investigate the effects of a highly bioavailable aglycone rich Red Clover isoflavone treatment to alleviate existing menopausal VMS, assessed for the first time by 24hour ambulatory skin conductance (SC)

**Methods and results:**

We conducted a parallel, double blind, randomised control trial of 62 peri-menopausal women aged 40–65, reporting ≥ 5 hot flushes/day and follicle stimulating hormone ≥35 IU/L. Participants received either twice daily treatment with bioavailable RC extract (RCE), providing 34 mg/d isoflavones and probiotics, or masked placebo formulation for 12 weeks. The primary outcome was change in daily hot flush frequency (HFF) from baseline to 12 weeks using 24hr SC. Secondary outcomes were change in SC determined hot flush intensity (HFI), self-reported HFF (rHFF) and hot flush severity (rHFS), blood pressure and plasma lipids. A significant decrease in 24hr HFF (P < 0.01) and HFI (P<0.05) was found when comparing change from baseline to 12 months of the RCE (**-4.3** HF/24hr, CI -6.8 to -2.3; **-12956** μS s^-1^, CI -20175 to -5737) with placebo (**0.79** HF/24hr, CI -1.56 to 3.15; **515** μS s^-1^, CI -5465 to 6496). rHFF was also significantly reduced (P <0.05)in the RCE (**-2.97** HFs/d, CI -4.77 to -1.17) group compared to placebo (**0.036** HFs/d, CI -2.42 to 2.49). Other parameters were non-significant. RCE was well tolerated.

**Conclusion:**

Results suggest that moderate doses of RCE were more effective and superior to placebo in reducing physiological and self-reported VMS. Findings support that objective physiological symptom assessment methods should be used together with self-report measures in future studies on menopausal VMS.

**Trial registration:**

ClinicalTrials.gov NCT02028702

## Introduction

Menopause symptoms severely reduce the quality of life of women worldwide, up to 80% of women may experience symptoms and it is estimated that in 2030 the at risk groups of peri- and post- menopausal women will reach 1.2 billion globally [1]. The core symptoms are hot flushes (HF) and night sweats (NS), collectively referred to as vasomotor symptoms (VMS); sleep disturbance and other secondary symptoms often also present [2].

These symptoms are largely a consequence of natural endogenous estrogen decline and dysregulation during peri- and post- menopause; estrogen deficiency is further associated with increased risk of osteoporosis, cardiovascular disease (CVD) and negative changes to lipid profile [3–5]. Hormone therapy (HT) is the current gold standard treatment for VMS. However substantial evidence supports that therapy increases cancer risk in estrogen receptor (ER) α rich tissues (e.g. uterus, breast and ovaries) [6,7]. The IMS and Revised Global Consensus Guidelines recommend and agree that both estrogen only and combined treatments: increase cancer risk with longer duration of use, that physicians should undertake a case by case risk benefit assessment prior to treatment, that treatments be limited to 5 years, that cancer risk is affected by time from the start of menopause and initiation of HT treatment, that the lowest effective dose be given, that patients undertaking HT have annual monitoring and that HTs are unsuitable for use in breast cancer operated patients/survivors suffering from estrogen deficient VMS [8–10]. Currently, there are no recommendations regarding ovarian cancer risk, a recent meta-analysis of 52 studies has shown equivalent relative risk increases of 1.43 and 1.37 for both estrogen only and combined therapies used for < 5 years respectively [11]. Considering that the median total VMS duration is shown to be 7.4 years and that the current guidelines for HT focus on minimising dose and duration of use, safely utilising HT to treat for the duration symptoms manifest is challenging [12]. Moreover, upon cessation of HT symptoms often return with greater severity and frequency [13]. As such it is of clinical and scientific importance to identify and develop effective treatments with minimal -effects that are suitable for consistent long term use.

Selective stimulation of ERs can be harnessed in order to minimise negative side effects induced by HT whilst simultaneously proffer beneficial effects for tissues requiring regulation by estrogen in order to function optimally. Isoflavones selectively modulate ERs as they retain a strong binding affinity to ERβ and weak affinity to ERα [14]. Estrogen binds strongly to both ER transcription factors (α and β) acting as a potent agonist [15]. Through ER β selectivity isoflavones proffer beneficial effects while minimising cancer risk; as ER β is highly expressed in non-gonadal tissues, such as adipose, brain, bone (osteoblasts), bone marrow, endothelial cells, kidney, intestinal mucosa, liver and lung parenchymal cells [14,16].A recent study investigating the effects of isoflavones on RNA sequencing in human breast cancer cell lines expressing ERα, ERβ or both ERs showed that various isoflavones shared similar gene expression pattern to estrogen; it was also found that isoflavones had distinct effects on gene expression and that isoflavones tended to exert more pro-apoptotic and less proliferative gene expression profile compared to estrogen [17].

Bioactive isoflavones from Red Clover (RC) (particularly Biochanin A and Formononetin) show promise as candidates for treatment of menopause symptoms as recent clinical trials have demonstrated beneficial effects against menopausal VMS and show minimal side effects with treatment [18–20]. Moreover, the safety profile of these compounds is promising and tested in numerous human trials. The longest clinical trial to date investigating high dose treatments (80mg/d and 120mg/d) for three years found no significant effects on adverse events or side effects compared to placebo [21]. Isoflavones have 1000 fold weaker binding affinity than estradiol to ERα [22]. Although the stronger binding affinity to ER β of isoflavone is also lower than estradiol, isoflavones can circulate at concentrations of 10,000 times that of estrogen and thereby achieve greater binding potential through abundance [23]. In plant isoflavones predominantly appear as glycosides, isoflavone glycosides represent a barrier to isoflavone uptake as they often violate at least one or more of Lipinski’s rule of 5 for permeability and oral drug-likeness [24,25]. To facilitate absorption, hydrolytic conversion by of glycosides by glycosidases to aglycone counterparts is necessitated. Recently, fermented isoflavone aglycone preparations have shown increased bioavailability compared to glycoside counterparts [26,27]. Utilisation of enzymatic techniques and probiotics have been demonstrated to increase the uptake of these compounds and may thereby improve efficacy of isoflavone treatments [28].

Previous investigations into menopausal VMS have exclusively relied on subjective self-report measures (i.e. Greene Climacteric Scale, Kupperman Index, Quality of Life Questionnaires and Hot flush frequency/severity diaries), these have been demonstrated to be plagued with bias and to be susceptible to placebo effect [29]. HFs are preceded by small increases in core body temperature and the subsequent sweat response during a HF event provides a surrogate objective physiological marker of menopausal hot flush frequency and intensity [30,31]. The Q sensor from Affectiva^™^ has been validated for determining the electrodermal activity storms healthy adults during sleeping at the wrist, these range from 0.005–0.05 μS [32]. Considering that the conventional criterion for a HF event is a ≥2μS rise in SC over 30 seconds, the Q sensor from Affectiva^™^ is well suited for hot flush capture [33]. Equivalent monitor sensitivity and performance sampling at the sternum and forearm has previously been demonstrated [34]. The use of skin conductance (SC)s in RCTs remains scarce due to the relative infancy of the methodology [18,35].

The present study randomises peri-menopausal women suffering from daily VMS into either treatment (with bioavailable RC derived isoflavones and probiotics) or placebo for a 12 week intervention period. The primary aim of the study was to determine the tolerability and efficacy of the RC and probiotic treatment in reducing hot flush frequency (HFF) relative to placebo, where physiological HFF is assessed for the first time using 24hr ambulatory SC coupled with 24 hr HFF diaries. We hypothesised that RC treatment would to a greater extent decrease HFF compared to placebo; secondarily we determined effects on VMS severity, GCS, fasted plasma lipids and blood pressure (BP) compared to placebo.

## Methods

### Study design

The trial was executed in accordance with guidelines laid down in the Declaration of Helsinki and approved by The Danish Ethical Committee (nr 1-10-72-487-12) and the Danish Data Protection Agency. In line with the International Committee of Medical Journal Editors the study protocol was registered at ClinicalTrials.gov (NCT02028702), along with all other ongoing and related trials.

**Fig 1:**
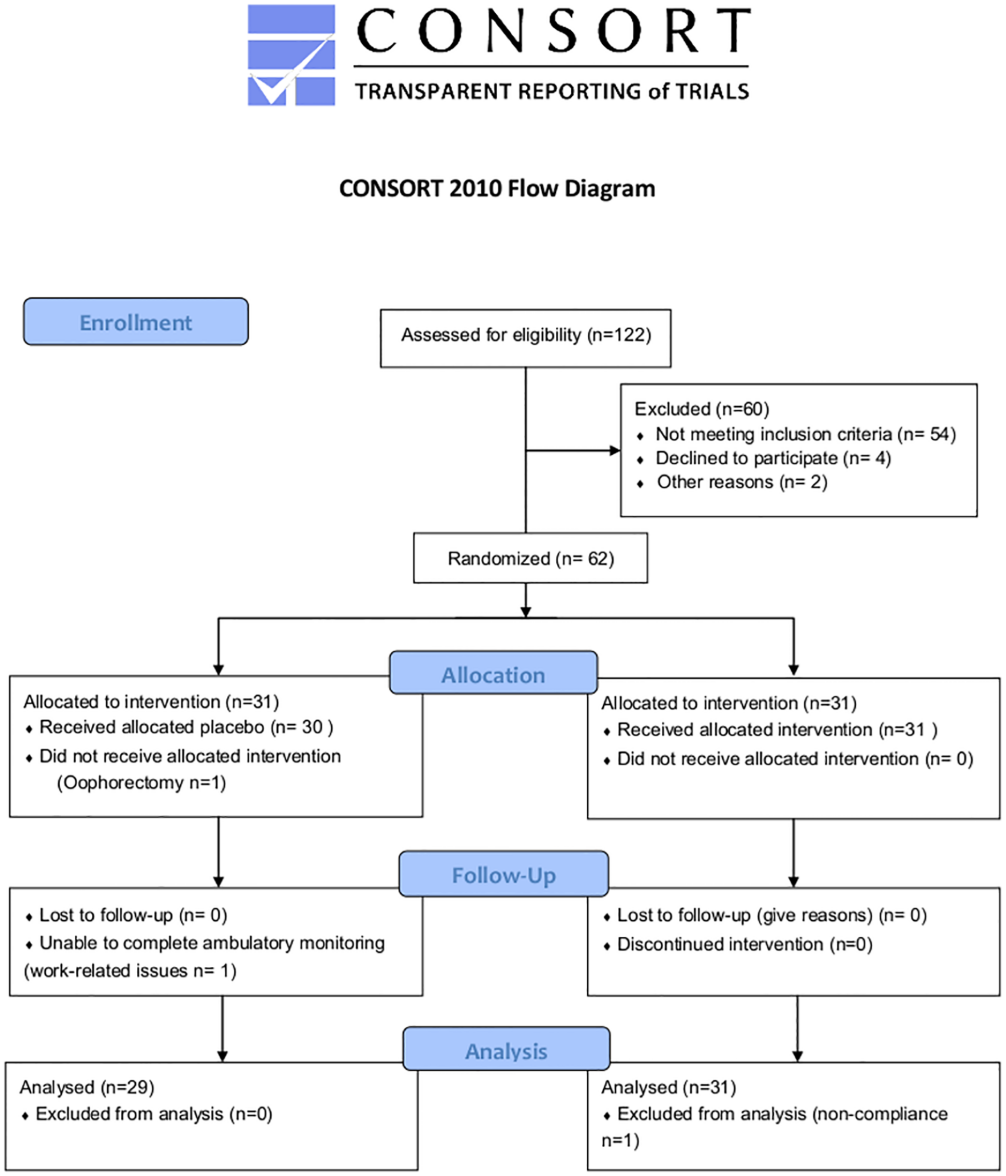
Consort diagram. In total 122 women were screened, 62 women met the inclusion criteria and were enrolled in the study. One participant dropped out due to personal reasons, two participants were excluded; one post medical examination due to undisclosed information regarding her physiological status and one submitted inadequate SC data due to lack of compliance during SC testing.

This is a double blind, randomised, placebo-controlled 12 week-trial. During screening (at -2 weeks) eligible participants provided informed written consent. Post briefing participants completed a full medical examination where baseline values were collected (BMI, age, self-reported HF frequency, FSH level, habitual and medical status). At baseline (week 0), participants were randomly allocated 1:1 into either RC (n = 31) or placebo groups (n = 31) by computer generated code (Fig 1). At weeks 0 and week 12, fasted blood and urine samples were taken and analysed, along with 24 hr ambulatory BP. The following day participants were requested to fill out a GCS, 24hour HFF diaries and undertake 24hr SC. Both 24 hour capture of SC and BP were carried out on separate days. Participants were briefed and empty RCE containers were collected and exchanged (accounting for losses) for compliance at weeks 3, 6, 9 and 12.

### Participants

The study enrolled 62 peri-menopausal women (FSH >35IU L^-1^) with existing menopause symptoms (≥ 5/day) administered a twice daily dose of either isoflavone and probiotic rich RC extract (75ml twice daily) or an equivalent placebo formulation. The peri-menopausal status of participants was defined in accordance with the criteria specified by the Stages of the Reproductive Ageing Workshop [36]. Participants were recruited from the local population from the Northern Denmark Region, referred either by local General Practitioners or contacted the research team in response to advertisements, posters and leaflets. Potential candidates were initially interviewed over the telephone. To be considered for the screening visit, participants were required to report ≥5 hot flushes/day, be aged between 40–65 years and have a BMI 20–40. Participants were screened from March to May 2012 and enrolled from June to study completion in September 2012.

The inclusion criteria were as follows: women with self-reporting menopausal symptoms (> 5 hot flushes per day); 40–65 years of age; BMI 20–40 kg/m^2^; reporting a variable cycle length of > 7 days different from normal, and having an FSH level ≥35 IU/L. The exclusion criteria were as follows: participation in other clinical trials within the last 6 months; cardiovascular-, chronic liver-, thyroid or kidney diseases; a history of cancer; a disease or condition that could influence the participants’ ability to follow the study protocol; alcohol or drug abuse; use of hormonal contraceptives within the last 3 months; excessive dietary intake of isoflavone rich foods; use of hormone therapy in the past 6 months; BP ≥160/110 mmHg; oophorectomy or amenorrhea > two years. Habitual medicine and supplement intake was registered prior to inclusion in the study. Eligibility or exclusion was assessed by the research team and medical professionals during the screening visit.

### Formulations

A heterogeneous culture of probiotic lactic acid bacteria (the exact composition of which is proprietary) was added to the RC extract to facilitate cold fermentation and improve bioavailability. Standardisation of post-fermentation aglycone content was validated by Liquid chromatography and Mass Spectrometry (LC-MS) by DB Lab A/S, (Odense, Denmark).

To mask taste and appearance characteristics of RCE, stevia and a natural sugar free raspberry/orange flavouring was added. Specifically, 90 litres of either water (placebo) or RC extract were sweetened with 18g stevia and 6.3 litres of sugar free raspberry/orange flavouring. The placebo was a water-based formulation in which 90 litres of water was mixed with 250g kavli brun kulør (brown food colouring, ammoniated caramel) to achieve likeness in appearance to RC extract. The RC and placebo extracts were packed in identical sealed brown cardboard cartons and marked with a red or blue code, corresponding to either the red or blue groups. All participants returned empty containers, which were collected by the research team and recorded to monitor compliance (set to 90%). Over the duration of the study an approximate total of 13.5 litres was consumed over 90 days. The RCE was taken with habitual morning and evening meals. All participants and the research team were blinded and had no knowledge of the content of the boxes throughout the course of the study.

### Isoflavone quantitation and validation by LC-MS

The post-fermentation isoflavone composition and quantitation analysis of the RCE was performed by DB Lab A/S (Odense, Denmark). Quantitation was assessed using high performance liquid chromatography with ultraviolet and mass spectrometry detection. Briefly, a Summit^®^ LC/MS (Sunnyvale, CA, USA) system consisting of a quaternary pump (P680 LPG), autosampler (ASI 100T), column oven (TCC-100) UV detector (PDA-100) and MS detector (Surveyor MSD Plus), all from Dionex, were used to perform the analysis. Four standards of the primary isoflavones (genistein, daidzein, formononetin and Biochanin A) and two glycoside derivatives (Ononin and Sissotrin) were obtained from Sigma Aldrich Denmark A/S (Brødby, Denmark). The RC extract was diluted 10 times (50ml to 5ml) by methanol prior to analysis.

Analysis (S1 Table) revealed that the majority of the isoflavones (≥90%) were converted to aglycones, although total conversion was not achieved (verified by the presence of ononin and sissotrin glucosides). In accordance with the DBlab analysis the participants who received active treatment were given a dose of 37.1 mg/d (of which 33.78 mg/d were aglycones). Due to lack of available standards other isoflavone glycosides were not included within the analysis.

### Skin conductance

**Fig 2:**
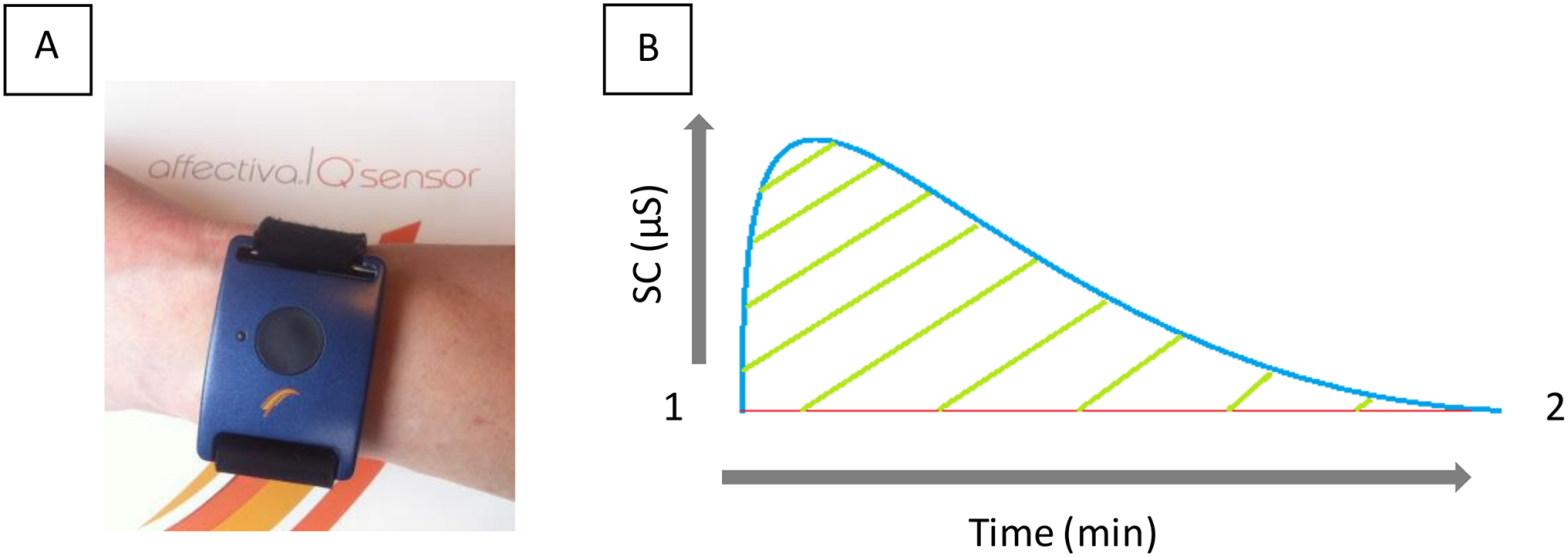
A and B: The model, program and SC monitor used for determination of hot flush events. (A) The Q sensor from Affectiva^™^ (Waltham, MA, USA) used for ambulatory monitoring and capture of participant electrodermal activity. It is applied to the wrist (in a similar fashion to a watch) allowing integrated dual electrodes contact with the ventral side of the arm. Electrodes detect changes in SC, facilitating the determination of changes in the sweat secretion of participants in micro Siemans (μS). (B) Example of an archetypal hot flush shape detected using SC with a “sharp rise and swishy tail” according to *Carpenter et al (1999)*.

Ambulatory 24 hour measurements of participants’ SC were taken at weeks 0 and 12. SC was measured using the Q sensor from Affectiva^™^ (Waltham, MA, USA) that enables measurements of electrodermal activity outside the laboratory (Fig 2A and 2B). Throughout the test period all participants consented to avoid all strenuous physical activity and to refrain from bathing to maintain basal sweat secretion. All participants gave consent to adhere to guidelines of use. Verification of data quality and duration was carried out using Affectiva Q software^™^ (Waltham, MA,USA), any data falling short of 22 hours, plagued with artefacts (such as a saw tooth shape or elevated baseline sweat secretion) or segmented by signal loss more than once, was repeated.

**Fig 3:**
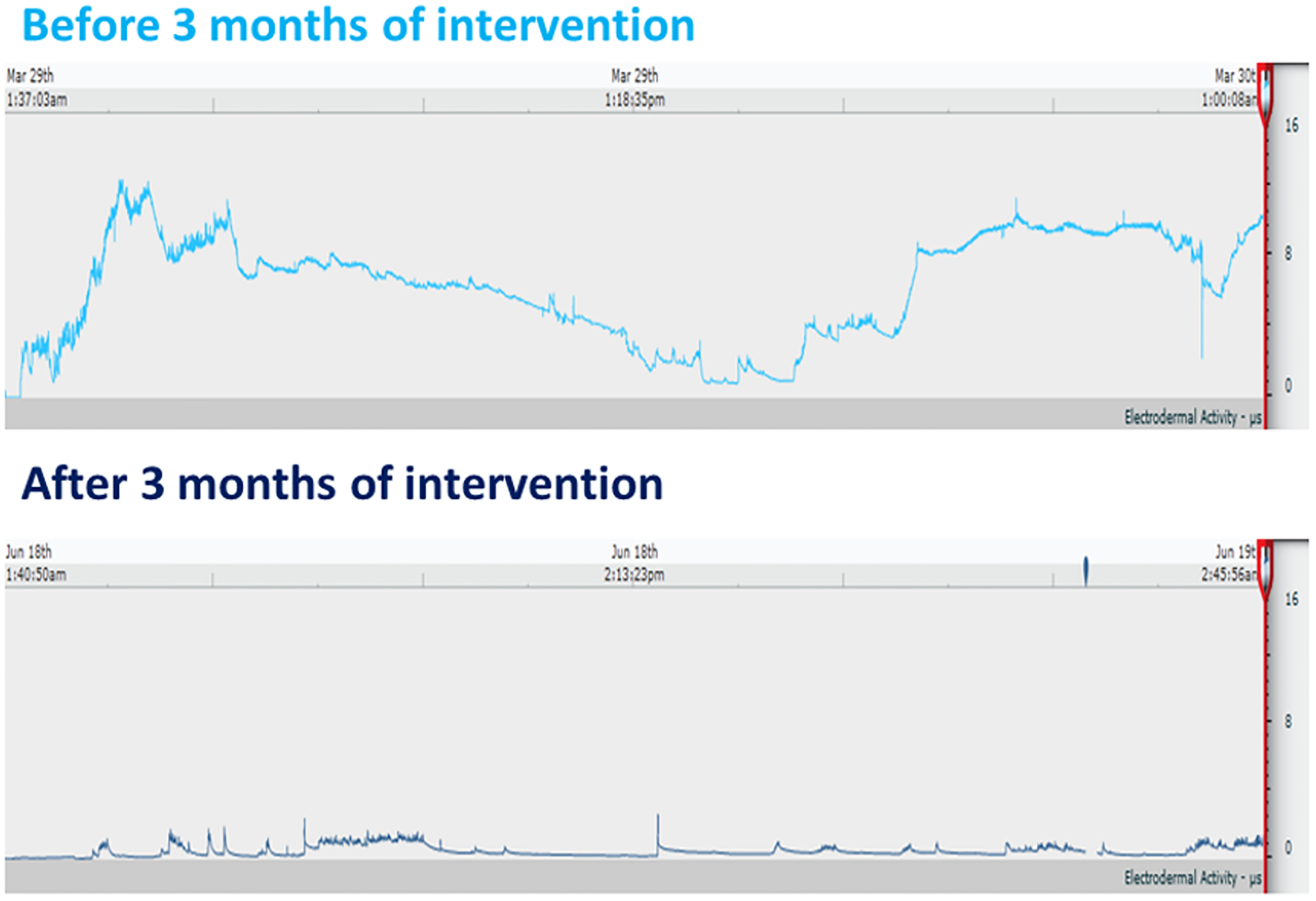
Typical skin conductance data sets. Examples of 24 hour skin conductance data sets, showing baseline (top) and post 12 week red clover treatment (bottom) data from a symptomatic menopausal participant.

HFs often last between 1–5 minutes but can reach durations of up to 1 hour [37]. In this study a HF event was defined as a ≥2μS rise over 30 seconds, actual hot flush duration was variable and cut off when either the peak was in decline and/or plateaued without further increases for at least 10 min and/or the conductance fell below the initial SC level any time after 30 seconds from the initiation of a HF event. Further ≥2μS SC increases were considered new hot flush events in the rare cases where SC had previously been in plateau and/or declining for extended periods above basal level post peak cut-off (Fig 3). Basal sweat secretion was individualised and variable.

### Hot flush analysis

HFF and HFI were assessed using MATLAB (Natick, MA, USA). A bespoke program (menopauseGUI.) was devised and developed by PhD Student. Esben Hansen, Aarhus University, using Matlab software. All SC datasets were standardised to 22 hours and imported into MATLAB. The menopauseGUI output for a total of 118 data files were individually approved and evaluated by two researchers. HFI was calculated as cumulative area under the curve from basal sweat secretion of each HF event. Ambulatory SC testing took place at weeks 0 and 12 of the study, over this period the average temperature in Northern Jutland rose on by 7.1°C from the start of the study to the end of the trial. Increases in temperature have previously been shown to elevate basal electrodermal activity and the magnitude of SC responses in controlled laboratory settings [38]. Post data collection HFI values were adjusted by a seasonal variation factor. This factor corresponded to the various individual increases in baseline basal sweat secretion of each participant.

### Green Climacteric Scale

A modified version of the original GCS (translated to Danish) previously described and validated in-house by Vestergaard et al (2003) was filled in on site by participants at week 0 and 12 under the guidance of a researcher [39]. Questionnaires at week 12 were either completed simultaneously with other tests or sent to the home address of a participant with a return envelope. All 21 items from each completed questionnaire were separated into total (1–21), vasomotor (questions 19–20), somatic (12–18), psychological (questions 1–11) and sexual groupings (21). For the purposes of this study depression and anxiety characteristics were not included.

### Analysis of plasma lipids

Blood plasma lipids were analysed using Dimension Vista analyser (Dade Behring, Newark, NE) that combines four technologies (i.e. photometry, nephelometry, V-LYTE integrated multisensory potentiometry, and LOCI chemiluminescence). Total cholesterol (TC), high-density lipoprotein cholesterol (HDL-C) and triglyceride (TG) concentrations were measured on using immunonephelometry assay. Final LDLC levels were calculated using the Friedewald equation, i.e. LDLC = TC—HDL—VLDL (estimated as triglycerides/50). All plasma samples were kept at– 80°C, (Sygehus Vendsyssel, Department of Clinical Biochemistry, Hjørring, Denmark) until analysis.

### Ambulatory blood pressure

BP measurements were taken at weeks 0 and 12 using the SpaceLab monitor (SpaceLabs Medical, Redmond, WA) and data assessed by SpaceLabs (Redmond, WA, USA) software. Mean 24hour arterial BP, heart rate, systolic and diastolic BP were taken at specified time intervals. Participants were required to abstain from medium and heavy exercise for the duration of 24 hour testing and to note down when they woke up and went to sleep.

### Statistics

Statistical analysis included all participants who completed the study. Data were analyzed using StataIC statistical software (version11.2, College Station, Texas, USA). Graphs were created by GraphPad Prism version 4 (GraphPad Software Inc., San Diego, CA, USA). All data were tested for normal distribution by way of visual inspection (QQ-plots, histograms and boxplots) and by D'Agostino-Pearson test. Differences in baseline and inter-group change over the duration of the study were determined for each parameter by way of Unpaired Student T-tests. Paired student T-tests were utilised to assess intra-group differences from baseline to 3 months for all groups and parameters.

Data are presented as mean and 95% confidence interval (CI); P <0.05 was considered significant. The A priori study power calculation was based on our primary endpoint of HFF. The number of participants determined by power calculation was 62 (31 in each group), with an expected drop-out rate of 15%, study power of 80%, SD of 3.3, a level of significance of 5%, with an expected effect size of 2.66. In accordance with the calculation it was necessary for 50 participants to complete the study. Minimal clinically important difference in hot flush frequency was found to be a difference of at least 15% as determined from data published by Lipovac et al (2012)[18].

## Results

### Participant characteristics

The baseline characteristics of participants are shown in Table 1. All data were normally distributed and there were no significant differences between the groups for any of the baseline parameters. FSH was indicative of menopause and both groups experienced more than 5 HF events/day.

**Table 1:** Baseline characteristics.

	Treatment (n = 30)	Placebo (n = 29)	*P value*^a^
**Age (years)**	52.40 (±4.64)	52.28 (±2.24)	0.90
**BMI (kg m^-1^)**	26.02 (±5.38)	25.45 (±3.34)	0.63
**FSH (mean, IU/l)**	73.70 (±27.10)	72.07 (±26.13)	0.82
**Hysterectomy (mean)**	0.10 (±0.30)	0.10 (±0.30)	0.97
**Smoker (mean)**	0.10 (±0.30)	0.21 (±0.41)	0.26
**HFF baseline (24hr peak no.)**	18.53 (±7.09)	15.93 (±7.02)	0.16
**HFI baseline (24hr μS s^-1^)**	32520 (±20966)	25590 (±28971)	0.30
**rHFF baseline (24hr peak no.)**	9.5 (±6.44)	8.55 (±6.87)	0.59
**rHFS baseline (24hr Score)**	17.30 (±12.98)	17.41 (±16.88)	0.98

Abbreviations: BMI, body mass index; FSH, follicle stimulating hormone; HFF, Hot Flush Frequency; HFI, Hot Flush Intensity; rHFF, self-Reported Hot Flush Frequency; rHFS, self-Reported Hot Flush Severity.

Data are summarised as mean (± SD).

^***a***^ P values for intergroup differences in baseline parameters determined by unpaired T test.

### Adherence to the study and adverse effects

A total of 59 women successfully completed the trial. RC treatment was well tolerated and there were no side effects and serious or adverse events in each group.

### Primary outcome: Physiological VMS

**Fig 4:**
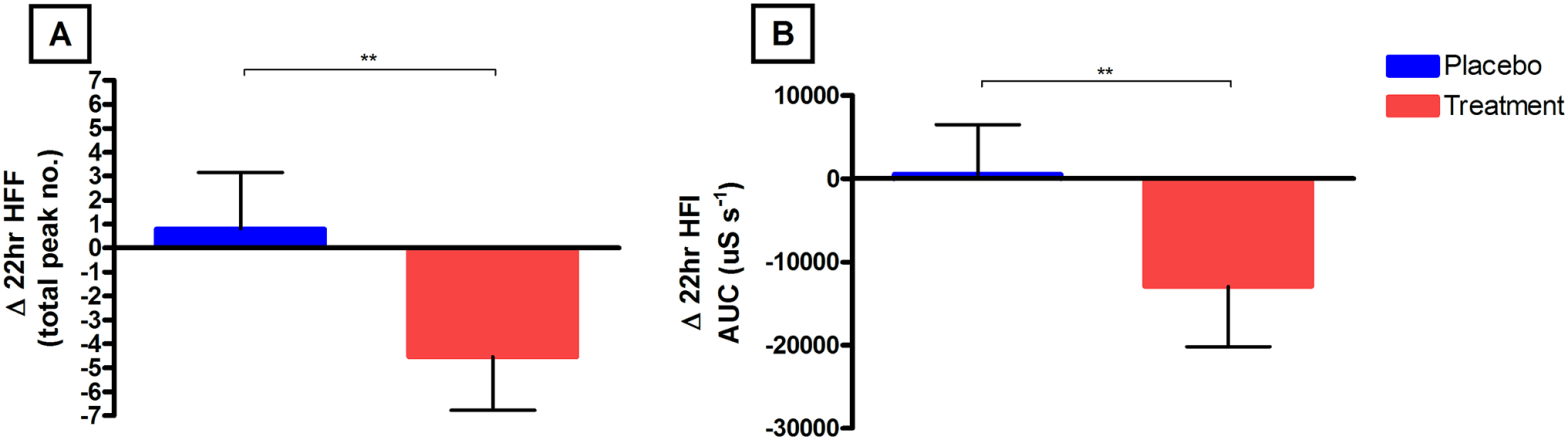
A and B: Change in SC determined 24hr HFF and HFI. (A) The mean (95% CI) change from baseline to 3 months in HFI (μS s-1) between the placebo and treatment groups. (B) The mean (95% CI) change from baseline to 3 months in 24hr HFF (total number of peaks) in the placebo and treatment groups. In all figures the Placebo group are marked as blue and RCE as red and significance is denoted as:* P<0.05, ** P<0.01.

A statistically significant (P< 0.01) decrease in physiological HFF from baseline to 3 months was found in the treatment group (Δ -4.3, CI -6.8 to– 2.3) compared to placebo (Δ0.79, CI -1.56 to 3.15). Physiological HFI also was significantly (P<0.01) reduced when comparing change from baseline to 12 months of the treatment (-12956 μS s^-1^, CI -20175 to -5737) group to placebo (515 μS s^-1^, CI -5465 to 6496); Fig 4A and 4B.

### Secondary outcomes: Self-reported VMS

**Fig 5:**
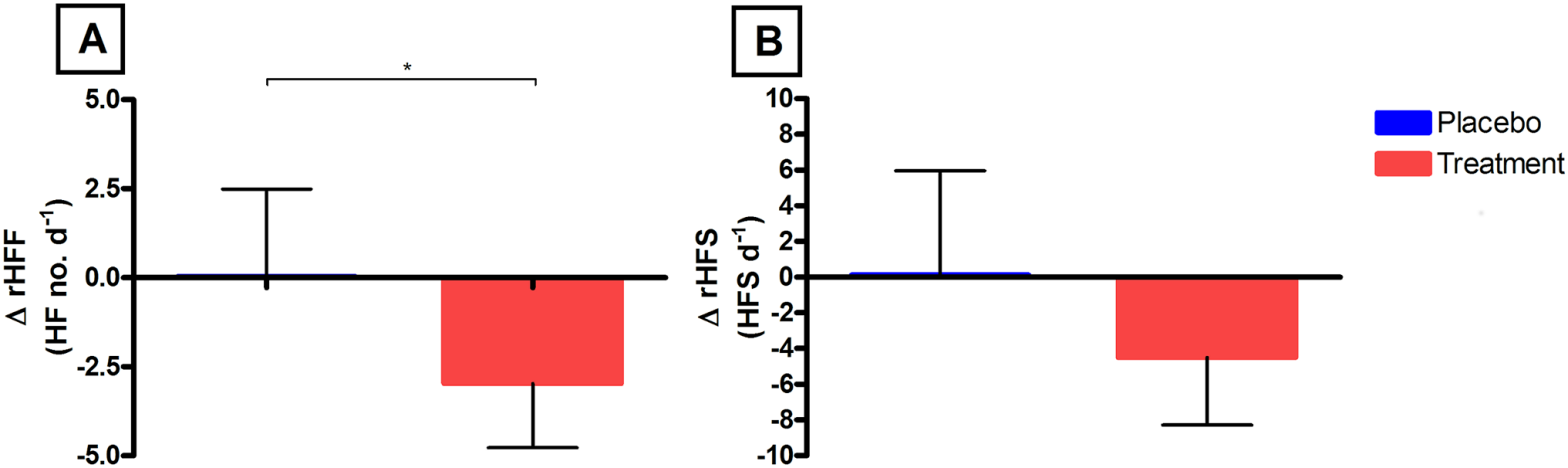
A and B: Self-reported rHFF and rHFS diaries. (A) The mean (95% CI) change from baseline to 3 months in rHFF in the placebo and RCE groups. (B) The mean (95% CI) change from baseline to 3months in 24hr rHFS in the placebo and treatment groups. In all figures the Placebo group are marked as blue and RCE as red and significance is denoted as:* P<0.05, ** P<0.01.

Self-reported HFF showed a significant (P< 0.05) reduction in the treatment group (-2.97 HF/d, CI -4.77 to -1.17) from baseline to 12 months in contrast to placebo (0.04, CI -2.42 to 2.49). There were no differences in change in rHFS between treatment and placebo groups (P = 0.18); Fig 5A and 5B.

### Green menopause scale

The 21 item GCS (Table 2) indicates no significant differences in the change in Total, Vasomotor, Somatic Psychological or parameters between treatment and placebo groups.

**Table 2:** Self-reported Greene Climacteric Scale.

**Group**	**GCS Baseline total** **(0–63)**	**GCS Endpoint total** **(0–63)**	***P* value**^a^	**ΔGCS Total** **(0–63)**	***P value***^b^
Placebo	20.82 ± 2.25	16 ± 1.61	<0.01	-4.82 (-8.17 to -1.46)	
Treatment	18.6 ± 1.32	14.75 ± 1.53	<0.05	-3.85 (-7.12 to -0.59)	0.67
**Group**	**GCS Baseline VMS** **(0–6)**	**GCS Endpoint VMS** **(0–6)**	***P* value**^a^	**ΔVMS** **(0–6)**	***P value***^b^
Placebo	4.59 ± 0.31	3.82 ± 0.38	0.053	-0.78 (-1.56 to 0.01)	
Treatment	4.35 ± 0.26	3.3 ± 0.45	<0.05	-1.05 (-2.07 to -0.26)	0.65
**Group**	**GCS Baseline** **psychological (0–33)**	**GCS Endpoint** **psychological (0–33)**	***P* value**^a^	**ΔPsychological** **(0–33)**	***P value***^b^
Placebo	10.09 ± 1.41	7.73 ± 0.91	<0.05	-2.36 (-4.54 to -0.19)	
Treatment	8.7 ± 1.06	6.8 ± 0.98	0.12	-1.9 (-4.32 to 0.52)	0.77
**Group**	**GCS Baseline Somatic** **(0–28)**	**GCS Endpoint Somatic (0–28)**	***P* value**^a^	**Δ Somatic** **(0–28)**	***P value***^b^
Placebo	4.91 ± 0.74	3.41 ± 0.49	<0.01	-1.5 (-2.58 to -0.43)	
Treatment	4.4 ± 0.55	3.8 ± 0.57	0.30	-0.6 (-1.77 to 0.57)	0.24

Abbreviations: GCS, Green Climacteric Scale; VMS, Vasomotor Symptoms; Δ, change.

Data are summarised as mean (± SD) and change data as mean (95% confidence interval. Higher GCS score indicates greater impact of symptom clusters on quality of life. 21 item GCS data; “Total” includes all 21 questions, “VMS” contains questions 19–20,”Psychological” covers questions 1–11 and “Somatic” incorporates questions 12–18. Each question is scored 0–3.

^***a***^ Significant intragroup difference determined by Paired student T-test

^***b***^ Significant intergroup difference in change assessed by Unpaired student T-test

### Effects of isoflavone treatment on fasted plasma lipids and blood pressure

There were no significant differences between baseline systolic and diastolic BP, LDL, HDL, TC and Triglyceride concentrations between groups. Moreover there were no significant differences in change in BP parameters (S2 Table) or plasma lipids (LDL, HDL, TC or triglycerides) between groups (Table 3). The data pertaining to BP is previously published by Thorup et al in 2015 [40].

**Table 3:** Plasma lipid profile.

**Group**	**Baseline HDL** **(mmol/L)**	**Endpoint HDL (mmol/L)**	***P* value**^a^	**Δ HDL** **(mmol/L)**	***P value***^b^
Placebo	1.733 ± 0.097	1.645 ± 0.072	0.47	-0.09 (-0.21 to 0.035)	
RCE	1.762 ± 0.154	1.744 ± 0.160	0.94	-0.02 (-0.14 to 0.11)	0.41
**Group**	**Baseline LDL** **(mmol/L)**	**Endpoint LDL** **(mmol/L)**	***P* value**^a^	Δ **LDL** **(mmol/L)**	***P value***^b^
Placebo	3.404 ± 0.169	3.298 ± 0.127	0.62	-0.11 (-0.34 to 0.13)	
RCE	3.262 ± 0.159	3.049 ± 0.140	0.31	-0.21 (-0.60 to 0.18)	0.64
**Group**	**Baseline Total** **Cholesterol (mmol/L)**	**Endpoint Total Cholesterol (mmol/L)**	***P* value**^a^	Δ **Total Cholesterol** **(mmol/L)**	***P value***^b^
Placebo	5.633 ± 0.100	5.518 ± 0.134	0.67	-0.12 (-0.45 to 0.22)	
RCE	5.384 ± 0.192	5.127 ± 0.213	0.38	-0.26 (-0.77 to 0.25)	0.64
**Group**	**Baseline** **Triglycerides (mmol/L)**	**Endpoint** **Triglycerides (mmol/L)**	***P* value**^a^	Δ **Triglycerides (mmol/L)**	***P value***^b^
Placebo	1.178 ± 0.104	1.181 ± 0.093	0.98	0.004 (-0.15 to 0.15)	
RCE	1.196 ± 0.086	1.183 ± 0.108	0.92	-0.01 (-0.19 to 0.16)	0.88

Abbreviations: HDL, High Density Lipoprotein; LDL, Low Density Lipoprotein; Δ, change.

Indicating baseline, end point values and change in fasted plasma lipids.

Absolute data are summarised as mean (± SD) and change data as mean (95% confidence interval)

^***a***^ Significant intragroup difference determined by Paired student T-test

^***b***^ Significant intergroup difference in change assessed by Unpaired student T-test

## Discussion

A recent comprehensive systematic meta-analysis of 62 clinical trials investigating plant based therapies for menopausal VMS has identified key limitations in study designs; mainly a lack of standardisation and accurate dosing of therapeutic compounds, pervasive use of self-report measures and poor characterisation of participants, particularly menopause status [41]. This study is one of the first to address these issues by including objective SC capture of hot flushes; determining and standardising the concentration and molecular form of isoflavone components of the RCE; and by utilising a combination of methodologies to characterise participant status and eligibility.

This study supports that moderate doses of RC derived isoflavone aglycones combined with probiotics can reduce the physiological and reported symptoms of menopause. The extent of the effect of RC treatment differed for the physiological measures (HFF -23.05%; HFI -39.84%) compared to the reported measures (rHFF -30.69%; rHFS -25.47%). This is likely due to reporting bias, missing data and hence greater variability within the self-report data. To the authors knowledge this is the first study to capture hot flush events during participant sleep cycles.

No significant between group differences were apparent in any of the GCS parameters. Both placebo and treatment groups showed improved symptoms from baseline in various aspects of the Greene Climacteric Scale, as such no significant intergroup differences were found. The GCS results indicated a prevalent placebo effect in questions within the psychological and somatic categories. The category directly addressing VMS demonstrated a significant within group reduction in the RC group alone, although the placebo group was non-significantly decreased with a p value of 0.053. These findings were unsurprising, as it is well documented that menopausal women are susceptible to placebo effect; other research has previously documented placebo groups self-reporting VMS improvements ranging from 20–60% [42]. Placebo effect may also explain the high variance in the placebo group’s rHFS data (Fig 5B) contributing to the non-significant intergroup outcome for rHFS; interestingly all other quantitative measures for hot flush parameters achieved inter-group significance. Data from hot flush diaries and questionnaires are heavily reliant on the participants’ understanding of the definition of a hot flush event and how their symptoms relate to that definition, as well as their diligence and consistency in reporting throughout the trial. A meta-analysis of 43 randomised control trials using isoflavone preparations showed all trials solely used one or more of a variety of self-report measures. The authors determined that the high placebo response, inter-individual variation in absorption and metabolism of isoflavones and variation of isoflavone components in formulations most likely led to disparity in the results of the trials [43]. The present study has utilised standardised isoflavone contents and probiotics to compensate for inter-individual differences in metabolism by introducing the same bacterial strains to all participants in the treatment arm. The consumption of probiotics (particularly lactic acid bacteria) is demonstrated to modulate the bacterial composition, hydrolytic capacity and metabolism of bioactive compounds in the GI tract [44]. Moreover certain bacteria are capable of synthesising isoflavone metabolites, of note is equol that retains a higher binding affinity to ERs than it’s precursor daidzein and may have a greater potency [45]. The current study has also used SC determination of VMS to compensate for the drawbacks of self-report methods. Overall the findings support that isoflavones can be effective against menopausal VMS and that more meaningful data can be achieved when common limitations of previous studies are addressed.

Research suggests that dietary isoflavones may reduce BP in hypertensive whilst not affecting normotensive patients [46]. In this study isoflavones and probiotics had no effect on BP in healthy, normotensive and non-obese peri-menopausal women (see S2 Table.). Studies focused on hypertensive and/or hyperlipidiaemic menopausal participants may be more appropriate to test for effects on BP and lipids as clinical research using soy has previously documented beneficial effects in these groups [47]. Menopausal women are at increased risk of developing higher circulating LDL to HDL ratio, increased TC and triglycerides [48]. Research indicates isoflavones improve circulating HDL:LDL ratio, reduce TC and triglyceride concentrations; possibly through interaction with ERs, peroxisome proliferator-activated receptors, aryl hydrocarbon receptor or other mechanisms [49]. In this study no effects on lipids were found. Considering that the participants had TC, LDL, HDL and Triglyceride levels within normal ranges this may explain the lack of significant change in this trial. Hyper-lipidaemic and/or obese estrogen deficient populations would be more suitable to detect potential effects.

## Conclusion

The use of a plant-based RCE in combination with probiotics in this study was effective in reducing menopausal VMS. Previous studies fail to account for inter-individual differences in GI microbiota of their participants or the molecular form of the isoflavones provided. This has led to high variability in isoflavone bioavailability, bioactivity and efficacy in other clinical trials. Standardisation of isoflavone concentration in commercial preparations has consistently been demonstrated to be poor; the most recent quantitation trial including 15 commercial isoflavone supplements using matrix solid phase dispersion and HPLC-DAD, found significantly lower determined concentrations compared to labelled isoflavone contents in 9 of the products [50]. The present study supports that well controlled isoflavone aglycone formulations and probiotics are effective in reducing VMS with minimal side effects.

The prevalent placebo effect influencing the self-report measures in this study clearly underlines the importance of developing and implementing objective assessment methods for interventions targeting menopausal VMS. This study demonstrates the challenges of critically interpreting and evaluating intervention outcomes using self-report data alone, these methods should be used in conjunction with self-report methodology and in this regard ambulatory SC technology represents an invaluable and promising clinical assessment tool.

## Supporting information

S1 Table Isoflavone content of the extract. Modified from Thorup et al 2015 *Evidence-Based Complement*. *Altern*. *Med*. 2015;2015:1–11.[40].
(DOCX)

S2 Table 24hour ambulatory blood pressure. Table 2 showing intragroup baseline and end trial 24 hour ambulatory blood pressures. Data are presented as mean values (± Standard Error), modified from Thorup et al 2015 *Evidence-Based Complement*. *Altern*. *Med*. 2015;2015:1–11.[40].
(DOCX)

S1 File Consort checklist. (DOC)

S2 File MenopauseGUI. (M)

S3 File Protocol Danish Ethics Committee. (DOCX)

S4 File Protocol Danish Ethics Committee (in Danish). (DOCX)

## Abbreviations

BMI: body mass index
BP: blood pressure
CI: confidence interval
CVD: cardiovascular disease
ER: estrogen receptor
FSH: follicle stimulating hormone
GI: gastro-intestinal
GCS: Greene climacteric scale
HDL: high density lipoprotein
HF: hot flushes
HFF: hot flush frequency
HFI: hot flush intensity
HPLC: high performance liquid chromatography
HT: hormone therapy
LDL: low density lipoprotein
RC: Red Clover
RCE: Red Clover extract
rHFF: self-reported hot flush frequency
rHFS: self-reported hot flush intensity
SC: skin conductance
SD: standard deviation
TC: total cholesterol
VLDL: very low density lipoprotein
VMS: vasomotor symptoms
