# Supplementary material for: Combined Red Clover isoflavones and probiotics potently reduce menopausal vasomotor symptoms
Source: PLoS One. 2017 Jun 7;12(6):e0176590. doi: 10.1371/journal.pone.0176590 (PMC5462345; doi:10.1371/journal.pone.0176590)
Supplement: S1 Table — Modified from Thorup et al 2015 Evidence-Based Complement. Altern. Med. 2015;2015:1–11.[40]. (DOCX) [file pone.0176590.s001.docx]

| **Isoflavone** | **Concentration (mg/l)** |
| --- | --- |
| Daidzein | 7.9 |
| Genistein | 20.9 |
| Biochanin A | 45.1 |
| Formononetin | 95 |
| Ononin | 9 |
| Sissotrin | 7.9 |
| Dry Matter | 30.4 g/L |
