# Supplementary material for: Combined Red Clover isoflavones and probiotics potently reduce menopausal vasomotor symptoms
Source: PLoS One. 2017 Jun 7;12(6):e0176590. doi: 10.1371/journal.pone.0176590 (PMC5462345; doi:10.1371/journal.pone.0176590)
Supplement: S2 Table — Table 2 showing intragroup baseline and end trial 24 hour ambulatory blood pressures. Data are presented as mean values (± Standard Error), modified from Thorup et al 2015 Evidence-Based Complement. Altern. Med. 2015;2015:1–11.[40]. (DOCX) [file pone.0176590.s002.docx]

| Group | Baseline 24 hour BP: Systolic (mmHg) | Endpoint 24 hour BP: Systolic (mmHg) | *P value* |
| --- | --- | --- | --- |
| Placebo | 124.5 ± 2.21 | 122.1 ± 2.23 | >0.05 |
| Treatment | 125.0 ± 1.89 | 122.6 ± 2.17 | >0.05 |
| Group | **Baseline 24 hour BP: Diastolic (mmHg)** | **Endpoint 24 hour BP: Diastolic (mmHg)** | ***P value*** |
| Placebo | 78.2 ± 1.26 | 77.3. ± 1.43 | >0.05 |
| Treatment | 78.9 ± 1.36 | 77.1 ± 1.40 | >0.05 |
