# Supplementary material for: Combined Red Clover isoflavones and probiotics potently reduce menopausal vasomotor symptoms
Source: PLoS One. 2017 Jun 7;12(6):e0176590. doi: 10.1371/journal.pone.0176590 (PMC5462345; doi:10.1371/journal.pone.0176590)
Supplement: S3 File — (DOCX) [file pone.0176590.s005.docx]

**Effekten af rød kløver ekstrakt på kvinder i overgangsalderen**

**Lektor, PhD, Per Bendix Jeppesen; M.Sc.,PhD-studerende, Anne Catherine S. Thorup; M.Sc.- studerende Max N.T. Lambert, Diabetesforskningslab. C, Aarhus Universitets Hospital, Tage-Hansens Gade 2, 8000 Århus C**

# Baggrund

De første symptomer på overgangsalder er ofte at menstruationerne bliver uregelmæssige, hvilket skyldes, at ægløsningen ikke fungerer helt som tidligere. Hormonbalancen bliver forstyrret, hvilket først ses på det ene kvindelige kønshormon - progesteron. Progesteron er det hormon, som kun findes fra ægløsning til menstruation, og som forbereder livmoderen på at modtage et befrugtet æg. Det andet kvindelige kønshormon – østrogen – dannes i starten af overgangsalderen i normal mængde, men efterhånden falder mængden drastisk.

Overgangsalderen rammer kvinder mellem 40- og 65-års alderen, og er for hovedparten af kvinderne forbundet med symptomer som hedeture (HT), nattesved, søvnløshed og hjertebanken, hvilket er de primære, og oftest forekommende symptomer. En del kvinder oplever også sekundære symptomer såsom seksuel dysfunktion, nedtrykthed, bekymring, hukommelsestab, træthed, hovedpine, muskelsmerter og vægtforøgelse. Knogleskørhed, hjerte-kar problemer samt negative forandring i fedt sammensætningen er alvorlige konsekvenser af hormonændringer i forbindelse med overgangsalderen. [1].

Symptomer i forbindelse med overgangsalderen er mest bredt blandt vestens kvinder; et studie fra Nordamerika - The North America Study of Women’s Health Across the Nation (SWAN) har klarlagt, hvilke kvinder der er mest ramt af overgangsalderen. Resultaterne fra SWAN studiet foreslår at etnicitet og race er nøgle faktorer, når det gælder hyppigheden og alvoren af overgangsalder relaterede symptomer. Kvinder hårdest ramt af overgangsalderen er afrikansk-amerikanske (46.5 %) og latinamerikansk (49 %) kvinder, efterfulgt af hvide kvinder (37%). De kvinder, der er mindst ramt af overgangsalder symptomer er ifølge SWAN japanske-amerikanske (34 %) og kinesiske-amerikanske (29 %) kvinder. Studiet konkluderer at grunden til den store forskel i overgangsalder relateret symptomer skyldes at en asiatisk diæt indeholder større mængder af isoflavon indeholdende mad i særdeleshed soja [2]. Isoflavoner er phytoøstrogener, der ligner det kvindelige kønshormon østrogen. Isoflavoner kan have samme virkning som hormonet østrogen, men i mildere grad. I sojaen er det især isoflavonerne genistein og daidzein, der har vist at have positiv østrogen lignende virkning.

Der er mange [gener](http://www.netdoktor.dk/sunderaad/fakta/overgangsalder/symptomer_i_overgangsalder.htm) forbundet med overgangsalderen, hvilket skyldes den faldende hormonproduktion, og en hormonbehandling vil derfor kunne afhjælpe mange af disse gener. Det er ikke problemfrit, at starte en hormonbehandling, og undersøgelser har vist at der er øget risiko for kræftudvikling samt udvikling af hjerte-karsygdomme ved langvarige hormonbehandlinger. En alternativ til den kontroversielle hormonbehandling er den alternative behandlingsform med kosttilskud indeholdende phytoøstrogener. En kilde som rødkløver (RK) indeholdende store mængder phytoøstrogener såsom isoflavoner, lignaner og coumestans.

Phytoestrogens er vist sig at have positive effekter på alvorlige sygdomme relateret til overgangsalderen såsom brystkræft og kardiovaskulære risikofaktorer. Derudover ser det ud til at hjælpe med at opretholde knogle massedensitet og forbedre lipidprofilen (reduktion af LDL: HDL, lipoprotein A, total kolesterol og muligvis også reducere triglycerider). RK har et højt indhold af østrogenlignende isoflavoner biochanin A, Formononetin og i mindre grad Genestien og Diadzien. De to førstnævnte er forløbere for Genestien og Diadzien, som alle let absorberes over tarmen. Frie aglycones og isoflavon β-glycosider absorberes effektivt ind i blodet som frie aglycones eller konjugeret med gluconuronic syre. Disse isoflavoner menes at være ansvarlig for en østrogen effekt og reduktion i overgangsalderens patogen relaterede symptomer. Bekymringer om risici af daglige tilskud af RK har vist sig at være uvæsentlige. De fleste studier har gået på potentielle kræftfremkaldende egenskaber og tildels kardiovaskulære komplikationer på grund af tilstedeværelsen af anti-koaguleringsmidler coumarins, men en væsentlig større antal studier understøtter det modsatte. I 2009 blev det påvist, at daglig administration af 120 mg RK isoflavoner i løbet af 12 måneder er sikkert og inducerede ikke nogen øget udvikling for kardiovaskulær sygdomme eller kræft udvikling [3, 4, 5, 6].

Der er udført adskillige forsøg med dagligt indtag af phytoøstrogener. Et review og meta-analyse fra 2007 undersøger 17 randomiseret kontrol forsøg, hvor RK blev brugt til behandling af overgangsalder relaterede symptomer. Konklusionen er ikke endegyldig, da der både findes positive, ingen og negative resultater af dagligt indtag af RK hos kvinder i overgangsalderen. Forskellen tilskrives heterogenitet mellem studierne yderligere karakteriseret ved forskelle i stikprøvestørrelse, kostvejledning, hedeture definition, genetiske og kulturelle forskelle i befolkninger, og inddragelse af kun ét eller begge peri-menopausale og postmenopausale deltagere. En stor del af studierne er også noteret som værende finansieret af en producent og dermed måske ikke uafhængig. Desuden er en del studier baseret på selv-rapportering, hvilket kan være et problem i det placebogruppen, der ligeledes oplever en forbedring af symptomerne, også benævnt som placeboeffekten. Med hensyn til dosering, er det rapporteret, at variansen på metaboliske egenskaber af de enkelte isoflavoner er meget forskellige [7, 8]. Det der gør dette studie interessant er at der er anvendt en helt ny og patenteret fermitering proces, der vil øge mægnden af aglucone isoflavoner, der nærmere optages over mave term kanalen, hvorved effekten af forbindelserne øges.

Hedeture er defineret som en følelse af varme og rødmen med tilhørende udvidelse af blodkarene og fald i kroppens kernetemperatur (KT). KT i sig selv er ikke en sikker og effektiv måleenhed til bestemmelse af HT, men svedmængden under HT har vist sig at være en mere brugbar måleenhed. Mængden af sved er brugbar pga. det måles objektivt, men kombineres det med subjektiv selvrapportering er det en nyttig og ny metode til at vurdere effektiviteten af ​​RK tilskud til at reducere HT symptomer og andre lidelser. [9, 10, 11]

# Hypotese

Et dagligt højt indtag af rød kløver ekstrakt har positiv indvirkning på symptomer forbundet med overgangsalderen på grund af deres høje niveau af isoflavoner sammenlignet med placebo.

# Formål

Formålet er, at gennemføre et dobbelt blindet, parallelt, randomiseret 3-måneders studie på kvinder i overgangsalderen for at undersøge, om et dagligt indtag af rødkløver ekstrakt har en positiv effekt på overgangsrelaterede symptomer sammenlignet med en placebogruppe.

# Specifikke Formål:

1. At undersøge i hvilken grad rødkløver ekstrakt kan reducere hyppigheden og intensiviteten af hedeture, søvnforstyrrelser samt daglige og natlige svedeture.
2. At undersøge om en daglig dosis af RK ekstrakt kan reducere demineralisering af knogler og forøge densiteten af knogle mineraler (BMD) på kort sigt.
3. At undersøge effekterne af en daglig dosis af RK ekstrakt på blodtrykket, lipid- og stofskifteprodukt-profiler, inflammatoriske markører samt mængden af østrogen.
4. At undersøge optaget og tilgængeligheden af isoflavoner i blodbanen.

Mange parametre er vigtige, når der er tale omovergangsalderen, men det primære måleparameter vil være hyppigheden og intensiviteten af hedeture, søvnforstyrrelser samt daglige og natlige svedeture. Hertil bruges en 24-timers sved målingsapparatur (SM), der måler huden udskillelse af sved samt temperaturen. Dette kombineres med et selv-reporteringsskema.

# Studie Design

Rød kløver ekstraktet, der anvendes til forsøget, fremstilles af firmaet Agrotech Itd. For at øge bio-tilgængeligheden bliver kløver ekstraktet behandlet således at isoflavonerne omdannes fra en glykosideret form til en aglycone form (fraspaltet glucose enheder). *[figure 1]*


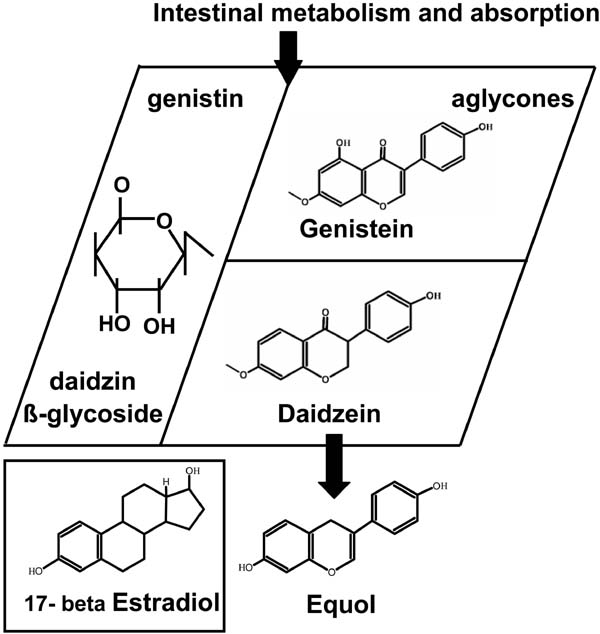


Figure 1:Omdannelse af glyconer (genistin og daidzin, betaglycoside form) til aglyconer (genistein og daidzein) ved enten sur hydrolyse eller bakteriel fordøjelse i en rotte model [12]

Kompositionen af isoflavoner og standariseringen af kosttilskudet er lavet af Agrotech Itd, og undersøgt ved hjælp af HPLC/MS før brug.

Studiet vil blive udført som et 3-måneders dobbelt blindet, parallelt, randomiseret studie, hvor 50 kvinder i overgangsalderen skal gennemføre studiet fordelt på 2 grupper. Forsøget skal forgå i foråret 2012 på Center for Klinisk Forskning, Sygehus Vendsyssel, under ledelse af Aarhus Universitets Hospital, Medicinsk Endokrinologisk Afd. MEA.

Forsøget vil før start blive anmeldt og godkendt af den Lokale Videnskabsetiske Komité i Region Midtjylland inkl. sekundære komité Region Nordjylland.

Forsøgsdeltagerne vil blive randomiseret i 2 grupper:

1. Test gruppe, der får rød kløver ekstrakt 2 gange dagligt.
2. En placebo gruppe, der får bitter saft (tranebær)

Deltagerne i gruppe 1 og 2 skal hver især dagligt indtage det udleverede ekstrakt (henholdsvis rød kløver ekstrakt og placebo), som uddeles på Sygehuset Vendsyssel, hvor der ligeledes fortages en samtale med deltagerne. Gruppe 1 får udleveret rød kløver ekstraktet, og gruppe 2 får udleveret samme mængde placebo.

Biomarkører af isoflavoner og estradiol i blodet vil blive brugt som komplians dvs. måle optagelse af kosttilskuddet. I forbindelse med studiet vil der blive etableret en forskningsbiobank og en biobank bestående af plasma- og urinprøver. Plasma- og urinprøverne nedfryses straks efter de er taget, og samtlige analyser i forbindelse med forskningsbiobanken analyseres umiddelbart efter forsøgets afslutning. Såvel deltagerne som datatilsynet vil blive informeret om og give skriftlig tilladelse til at prøverne gemmes i en forskningsbiobank og biobank. Prøverne i biobanken kan kun anvendes til forskning efter godkendelse fra videnskabetisk komité. Prøverne i forskningsbiobanken og biobanken vil blive mærket med deltagerinitialer, fødselsdato samt dato., og mappen med tilhørende identifikation, vil blive opbevaret et separat aflåst sted, således at det kun er de af forskergruppen autoriserede, der har adgang dertil.

Patienten vil i samtykkeerklæringen blive informeret om deres rettigheder vedr. indsamling til biobanken.

**Studiedesign**


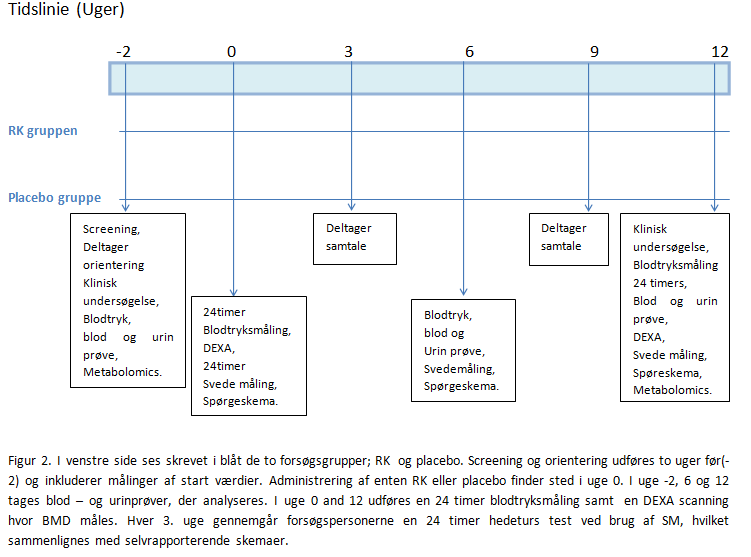


Forud for forsøget gives en mundtlig orientering. Hvis deltageren efter mindst en uges overvejelse stadig ønsker at tage del i forsøget og gennemgå screenings procedurer.

Kræft relateret sygdomshistorie, blod og antropometriske målinger (BMI, hoftemål og lipid profil) tages af en teknisk erfaren person. Deltagerne screenes for at udvælge dem, der opfylder forsøgskriterierne. De endelige forsøgspersoner får lavet 24-timers blodtryksmålingerne, hvorefter de randomiseres i to grupper (placebo og RK). Forsøgspersonerne fører dagbog over antallet af daglige og natlige hedeslag gennem den 3 måneder lange forsøgsperiode. Kupperman index spørgeskemaer benyttes til bedømmelse af intensiteten af symptomer og sendes ud ved uge 0, 6 and 12 (indeholdende returkonvolut og frimærke), og returneres af forsøgspersonen i slutningen af ugen.

Deltagere kontaktes ugentligt af forsøgsansvarlig (Lektor Phd. Per Bendix Jeppesen) eller en af de øvrige deltagere for at høre forsøgsdeltagerne om forsøgets forløb. Hver 3. uge møder deltagerne op på Sygehus Vendsyssel for at afhente dosis af placebo eller RK ekstraktet. Der er i alt 3 obligatoriske besøg, uge 0, 6 og 12, hvor der tages urin- og blodprøver til analyse (lipider, inflammatoriske markører og isoflavoner). Ved uge 0, 6 og 12 udføres ydermere en 24-timers måling af hudens svedsekretion og temperatur. Deltageren skal spise et standard aften måltid aftenen før samt morgenmad. Ved forsøgets start og slut udføres ligeledes en 24-timers blodtryks måling. Alle procedure vil blive beskrevet I SOPér (Standard Operating Proceedure ).

Overblik over studiets processer og tidspunker er vist i tabel 1, table 2 og table 3.

| Målinger (uger) | -2 | 0 | 3 | 6 | 9 | 12 |
| --- | --- | --- | --- | --- | --- | --- |
| Klinisk undersøgelse | X | X |  |  |  | X |
| Spørgeskema |  | X | X | X | X | X |
| BMI | X | X |  |  |  | X |
| DEXA |  | X |  |  |  | X |
| Blod og urin til metabolomics | X |  |  | X |  | X |
| Blodtryk og blodprøve (lipider og inflammatoriske markører) | X |  |  | X |  | X |
| 24 timer blodtryksmåling |  | X |  |  |  | X |
| 24 timers hudmåling af sved og temperatur |  | X |  | X |  | X |
| Blod test til vurdering af komplians | X |  |  | X |  | X |
| Samtale | X | X | X | X | X | X |

Tabel 1 *Behandling af deltagerne og generelt målinger af parametre*

| Blodprøve (Uger) | -2 | 0 | 3 | 6 | 9 | 12 |
| --- | --- | --- | --- | --- | --- | --- |
| Lipid Profil (tryglicerider, LDL:HDL, FFA og Total Kolesterol , ApoLp-a) | X |  |  | X |  | X |
| Isoflavone komplians (Genstien, Diadzien, Formononetin, Bichanin A) | X |  |  | X |  | X |
| Cytokiner (NF-κB ↓, IL6, TNFα, NOS and Cyc O 2: ↑IL 10) | X |  |  | X |  | X |
| Follicle Stimulating Hormone (FSH) (laves på alle , skal benyttes til inklusion kriterier) samt Estradiol | X |  |  | X |  | X |

Tabel 2 *Blodt prøve parameter: ,isoflavone og hormoner for kompliance, Lipider og Inflamation markør*

| Urinprøve (Uger) | -2 | 0 | 3 | 6 | 9 | 12 |
| --- | --- | --- | --- | --- | --- | --- |
| Metabolomics  HPLC/MS | X |  |  | X |  | X |

Tabel 3 *Metabolomics af urine prøve*

# Forsøgespersoner

50 forsøgspersoner i alderen 40-65 år skal gennemføre projektet i perioden forår 2012. Deltagerne skal holde deres fysiske aktivitet, ryge- og alkoholvaner stabile gennem hele forsøgsperioden. Brugen af blodtryks - og lipidsænkende medicin kan fortsættes i forsøgsperioden.

**Inklusionkriterier**:

- Alder 40-65 år, kun kvinder.
- Oplever daglige hedeture
- Body Mass Index (BMI) mellem 20-40
- Have uregelmæssig blødninger
- FSH niveauer mellem 30 - 50 mUI/mL

**Eksklusions kriterier**:

- Samtidig deltagelse i andre kliniske forsøg indenfor de sidste 3 måneder
- Udebleven blødninger i mere end 6 måneder
- Udtalt kardiovaskular-, psykiatrisk-, neurologisk-, og/eller nyresygdom.
- Alkohol- eller pillemisbrug samt akut sygdom.
- Blodtryk > 160/110
- Gravide og ammende kvinder

En deltager udelukkes fra studiet, hvis vedkommende gentagne gange ikke følger studiets retningslinjer.

#

# Rekruttering af deltagerne

Deltagerne rekrutteres af læger via ambulatoriet ved Sygehus Vendsyssel (kontakt med ambulatoriet i forbindelse med fremmøde af anden årsag), praktiserende læger i området samt via annoncer i lokalblade som Nordjyske, Vendelbo Posten (annoncen vedlagt) samt opslag, hvorefter mulige deltagere henvises til den forsøgsansvarlige Per Bendix Jeppesen. Deltagerne informeres kort mundtligt om projektet pr telefon. Er deltageren stadig interesseret udleveres/tilsendes en skriftlig deltagerinformation samt ”Forsøgspersoners rettigheder i et biomedicinsk forskningsprojekt” udarbejdet af Den Central Videnskabsetiske komité. Efter at deltagerne hjemme har haft mulighed for at læse den skriftlige deltagerinformation igennem, inviteres deltageren til en mundtlig samtale, hvor projektansvarlig vil kunne besvare eventuelle spørgsmål, og senere indhente samtykke. Der gives mindst en uges betænkningstid fra første mundtlige information til indhentning af samtykke. Deltagerne har mulighed for at medbringe en bisidder. Forsøgspersonerne oplyses desuden om, at deltagelse er frivilligt, og at de på ethvert tidspunkt kan trække sig fra undersøgelsen uden, at det får indflydelse på deres behandling eller kontrol på sygehuset. Deltagerne er forsikret gennem Sygehusets Patientforsikringen.

# Håndtering af forsøgsperson-relaterede oplysninger

Alle oplysninger om forsøgsdeltagerne beskyttes efter Lov om behandling af personoplysninger og Sundhedsloven (GCP regler). Alle prøver (blod og urin) vil blive kodet (deltager ID), så kun den forsøgsansvarlige/-centret kan forbinde resultaterne til den enkelte deltager. Resultaterne er fortrolige og udleveres ikke til udenforstående personer. Studiet anmeldes til Datatilsynet.

# Økonomiske forhold

Studiet, der gennemføres på Center for Klinisk Forskning, Sygehus Vendsyssel, under administration af Medicinsk Endokrinologisk Afd. MEA (Aarhus Universitets Hospital) finansieres af Agrotech Itd.

Initiativet til forsøget er lektor, Phd, Per Bendix Jeppesen, fra Medicinsk Endokrinologisk Afd. MEA (Aarhus Universitets Hospital).

Ingen af de medvirkende i studiet har økonomiske interesser knyttet til private virksomheder eller fonde der indgår i studiet. Projektet overholder Lov om behandling af personoplysninger, og projektet anmeldes til datatilsynet.

Der ydes ikke honorar til forsøgspersonerne for deltagelse. Der ydes godtgørelse for kørsels- og transportudgifter og tabt arbejdsfortjeneste efter statens takster i forbindelse med undersøgelserne på sygehuset.

#

# Risici ved deltagelse i studiet

Deltagerne bedes fortløbende om at registrerer alle bivirkninger, der kan relateres til rød kløver ekstraktet. Eventuelle handlinger for at reducere negative bivirkninger diskuteres imellem de involverede partnere, for at reducere disse.

Blodprøveudtagning er forbundet med en lille risiko for perforation af en vene og et efterfølgende lille, overfladisk hæmatom. Der foreligger desuden en minimal risiko for infektion ved indstikstedet.

Der er en mindre strålerisiko ved DEXA-skanningen, der i øvrigt er uden ubehag. For DEXA-skanningen udgør stråledosis 0.02-0.05 mSv pr. undersøgelse. Til sammenligning er den årlige baggrundsstråling ca. 3 mSv.

# Videnskabsetisk redegørelse

De risici og ulemper, som deltagelse i studiet kan udgøre for deltagerne er gennemgået i det foregående afsnit. Deltagelse i studiet vil have følgende fordele: en grundig klinisk- og biokemisk helbredsundersøgelse samt bestemmelse af knogledensiteten. Ved forsøgets afslutning informeres patienterne om undersøgelsens resultater. Såfremt blodprøverne udviser noget abnormt vil patienten blive rådet til kontrol ved egen læge eller henvist til relevant afdeling.

Studiet vil give ny værdifuld viden og dokumentation om rød kløver ekstrakt kan afhjælpe kvinder, der lider af overgangsalder relaterede symptomer. Denne viden kan herved blive et nyttigt værktøj i behandling af overgangsalderen.

Alt i alt vurderes studiet at være forbundet med minimale risici for deltagerne, at det vil bibringe deltagerne relevante oplysninger om egen helbredstilstand, og det vil give værdifuld biomedicinsk viden om rød kløver ekstrakt som et nyt kosttilskud. På den baggrund vurderes studiet forsvarligt og værd at gennemføre fra et videnskabelig og etisk perspektiv.

# References

1) J. Moilanen, A.-M. Aalto, E. Hemminki, A.R. Aro, J. Raitanen and R. Luoto (2010) Prevalence of menopause symptoms and their association with lifestyle among Finnish middle-aged women Maturitas: 67: 368–374

2) S. Palacios, V. W. Henderson, N. Siseles, D. Tan and P. Villaseca (2010) Age of menopause and impact of climacteric symptoms by geographical region. CLIMACTERIC 13: 419–428

3) Peter Chedraui, Glenda San Miguel, Luis Hidalgo, Nancu Morocho & Susana Ross (2008) Effect of Trifolium pratense-derived isoflavones on lipid profile of postmenopausal women with increased body mass index. Gynocological Endocrinology: 24 (11): 620-624

4) Stacy E. geller and Laura Studee (2006) Soy and red clover for midlife and aging. Climeractic: 9 (4): 245- 263

5) Joanna Thompson Coom, Max H. Pittler, Edward Ernst (2007) Trifolium Pratense isoflavones in the treatment of menopausal hot flushes: A systematic review and meta-analysis. Phytomedicine 14, 153-159

6) [Geller SE](http://www.ncbi.nlm.nih.gov/pubmed?term=%22Geller%20SE%22%5BAuthor%5D), [Shulman LP](http://www.ncbi.nlm.nih.gov/pubmed?term=%22Shulman%20LP%22%5BAuthor%5D), [van Breemen RB](http://www.ncbi.nlm.nih.gov/pubmed?term=%22van%20Breemen%20RB%22%5BAuthor%5D), [Banuvar S](http://www.ncbi.nlm.nih.gov/pubmed?term=%22Banuvar%20S%22%5BAuthor%5D), [Zhou Y](http://www.ncbi.nlm.nih.gov/pubmed?term=%22Zhou%20Y%22%5BAuthor%5D), [Epstein G](http://www.ncbi.nlm.nih.gov/pubmed?term=%22Epstein%20G%22%5BAuthor%5D), [Hedayat S](http://www.ncbi.nlm.nih.gov/pubmed?term=%22Hedayat%20S%22%5BAuthor%5D), [Nikolic D](http://www.ncbi.nlm.nih.gov/pubmed?term=%22Nikolic%20D%22%5BAuthor%5D), [Krause EC](http://www.ncbi.nlm.nih.gov/pubmed?term=%22Krause%20EC%22%5BAuthor%5D), [Piersen CE](http://www.ncbi.nlm.nih.gov/pubmed?term=%22Piersen%20CE%22%5BAuthor%5D), [Bolton JL](http://www.ncbi.nlm.nih.gov/pubmed?term=%22Bolton%20JL%22%5BAuthor%5D), [Pauli GF](http://www.ncbi.nlm.nih.gov/pubmed?term=%22Pauli%20GF%22%5BAuthor%5D) and [Farnsworth NR](http://www.ncbi.nlm.nih.gov/pubmed?term=%22Farnsworth%20NR%22%5BAuthor%5D). (2009) Safety and Efficacy of Black Cohosh and Red Clover for the Management of Vasomotor Symptoms: A Randomised Control Trial. [Menopause.](http://www.ncbi.nlm.nih.gov/pubmed/19609225) 16(6):1156-66.

7) V. Beck, U. Rohr, A. Jungbaur (2005) Phytoestrogens derived from red clover: An alternative to estrogen therapy? Journal of steroid biochemistry and molecular biology 94: 499 – 518

8) Marjus Lipovic, Peter Chedraui, Christine Gruenhut, Anca Gocan, Christine Kurz, Benedikt Neuber and Martin Imhof (2011) The effect of red clover isoflavones supplementation over vasomotor and menopausal symptoms in postmenopausal women. Gynocological Endocrinology 1-5

9) Heather G. Miller, MFS,PhD and Maria Li, MBA PhD (2004) Measuring Hot Flashes: Summary of a National institutes of Health Workshop. Mayo Clinic Proceedings 79: 777-781

10) J.G. Webster, D. E. Bahr, M. C. Shults, D. G. Grady and J. Macer (2004) A miniature sternal skin-attached hot flash recorder. National Centre for complementary and Alternative medicine, Bahr Management Inc.

11) Nelson HD, Haney E, Humphrey L, et al. (2005) Management of Menopause-Related Symptoms. Rockville (MD): Agency for Healthcare Research and Quality (US); (Evidence Reports/Technology Assessments, No. 120.)

12) Edwin D. Lephart, Kenneth D. R. Setchell, Robert J. Handa, and Trent D. Lund (2004) Behavioral Effects of Endocrine-disrupting Substances: Phytoestrogens. ILAR Journal 45 (4): 443-454
