# Supplementary material for: Combined Red Clover isoflavones and probiotics potently reduce menopausal vasomotor symptoms
Source: PLoS One. 2017 Jun 7;12(6):e0176590. doi: 10.1371/journal.pone.0176590 (PMC5462345; doi:10.1371/journal.pone.0176590)
Supplement: S4 File — (DOCX) [file pone.0176590.s006.docx]

**The effects of red clover extract on menopausal women**

**Associate Prof, PhD., PhD, Per Bendix Jeppesen; M.Sc.,PhD, Anne Catherine S. Thorup; M.Sc., PhD-student Max N.T. Lambert, Dept. of Clinical Medicine, Aarhus University Hospital, Tage-Hansens Gade 2, 8000 Aarhus C, Danmark**

English Resume: The aim of this study is to determine whether a daily intake of Red Clover derived isoflavones combined with probiotics can significantly improve physiological and psychological vasomotor symptoms in Menopausal women. Hormone replacement therapy is the current gold standard treatment for these symptoms but due to undesirable carcinogenic side effects its use is limited. Isoflavones can selectively modulate estrogen receptors. This characteristic can be harnessed to target tissues that require stimulation by estrogen to function normally and simultaneously minimise effects on estrogen sensitive tissues. Red Clover contains a unique profile of isoflavones (Formononetin and Biochanin A) that may exert greater effect than soy counterparts (Daidzein and Genistein). The hydrolytic capacity of the intestine is a key barrier to the bioavailability and bioactivity of isoflavone treatments, processing methods using lactic acid bacteria can be used to increase isoflavone uptake and effectiveness of isoflavone treatments. Isoflavones are ingested in Asia in concentrations between 30 -50 mg/d on average and research has shown that 1 year daily intake of 120mg/d of isoflavones to be safe with minimal side effects and as such have a promising safety profile.

Contents

[Background 1](#_Toc308428406)

[Hypothesis 4](#_Toc308428407)

[Aim 4](#_Toc308428408)

[Specific aims: 4](#_Toc308428409)

[Study Design 4](#_Toc308428410)

[Participants 8](#_Toc308428411)

[Recruitment of participants 9](#_Toc308428412)

[Handeling of participants data and data protection 9](#_Toc308428413)

[Publication 10](#_Toc308428414)

[Finansing of the study 10](#_Toc308428415)

[Risks with participation in the study 10](#_Toc308428416)

Research [Ethics 11](#_Toc308428417)

[References 12](#_Toc308428418)

# Hypothesis

A daily high intake of red clover extract will have positive effect on symptoms associated with menopause compared to placebo.

# Aim

# The purpose is to conduct a double-blind, parallel, randomized 3-month study on menopausal women, to determine whether a daily intake of red clover extract has a positive effect on menopausal vasomotor symptoms compared to placebo group.

# Specific Aims

1) To examine the extent to which red clover extract can reduce the frequency and intensity of hot flashes, sleep disturbances and daily and night sweats.

2) To investigate if a daily dose of RK extract may reduce demineralization of bone and increase the density of the bone mineral density (BMD) in the short term.

3) To investigate the effects of a daily dose of RK extract on blood pressure, and lipid metabolite profiles, inflammatory markers, as well as the amount of estrogen.

4) To study recorded and the availability of isoflavones in the blood stream.

Various parameters are important in the case of menopausal vasomotor symptoms, but the primary measurement parameter will be the frequency and intensity of hot flushes, sleep disturbances and daily and night sweats. Outcomes will be determined using 24-hour ambulatory skin conductance apparatus (SC), which measures the skin secretion of sweat and temperature. This will be combined with self-report methodologies.

# Studie Design

Red clover extract used in the trial is manufactured by Agro Tech Itd. In order to increase the bio-availability is clover extract is treated so that the isoflavones are converted from one form to a glykosideret aglycone form (split off glucose units). [Figure 1]


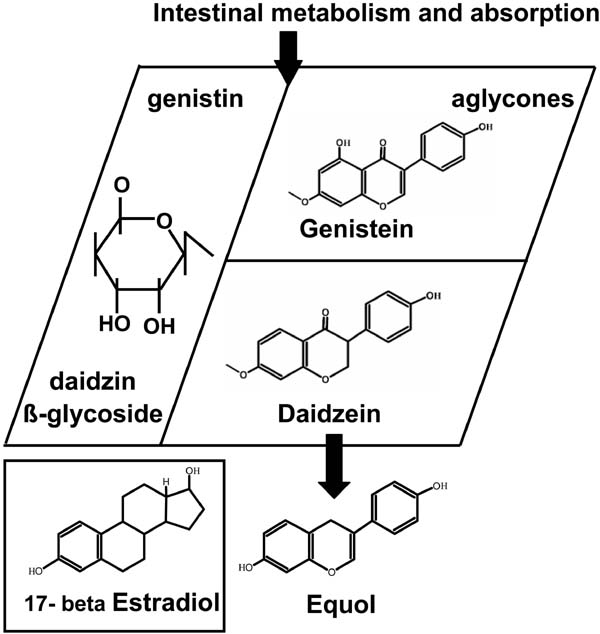


Figure 1: Conversion of isoflavone glycosides (genistein and daidzein, beta glycosidic form) to the aglycones (daidzein and genistein) by either acid hydrolysis or bacterial digestion in a rat model [12]

The composition of isoflavones and standardization of study formulations are made my AgroTech Itd, and examined by HPLC / MS prior to use.

The study will be conducted as a 3-month double-blind, parallel, randomized control trial in which 50 women in menopause are required to complete the study randomised into 2 groups. The trial will take place in spring 2012 at the Center for Clinical Research, Vendsyssel Hospital, led by Aarhus University Hospital, Endocrinology Department. MEA. .

The trial will start before being reviewed and approved by the Local Research Ethics Committee in Region Midtjylland incl. secondary committee North Jutland.

The subjects will be randomized into 2 groups:

1. Test group that gets red clover extract 2 times daily.

2. A placebo group who get bitter masked placebo

Participants in groups 1 and 2, each consume the given extract (respectively red clover extract and placebo) at the hospital Vendsyssel during a briefing with the participants. Group 1 will receive red clover extract, and Group 2 will receive the same amount of placebo.

Biomarkers of isoflavones and estradiol in plasma will be used as compliance i.e. measuring the absorption of the isoflavone components. During the study we will establish a research biobank consisting of plasma and urine samples. Plasma and urine samples are frozen immediately after they are taken, and all analyzes related to the research biobank analyzed immediately after completion of the trial. All participants will be informed and are required to give written permission for the samples stored in a research biobank. The samples in the biobank may only be used for research after approval by the research ethics committee. The samples of the research biobank and biobank will be labelled with participant initials, date of birth and the date of collection. A folder with corresponding sample identification details will be stored in a separate locked location.

During the pre-screening visit potential participants are required to provide written consent and will prior be informed about their rights regarding. collecting biobank and study participation.

**Study design**


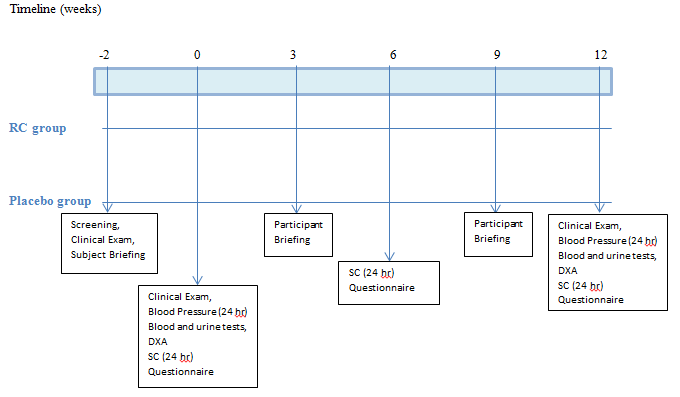


Figure 2 on the left side are the two groups treatment and placebo (below). Screening and participant orientation is carried out two weeks prior to starting in the study. Adminstration of either treatment or placebo takes place at week 0. During weeks 0,6 and 12 fasted urine and blood samples are taken and analysed. During weeks 0 and 12 24 hour ambulatory skin conductance, blood pressure and DEXA scans take place. Every 3 weeks participants are also briefed, receive new formulation and return empty extract containers.

The potential eligible participant is then given a week to decide if they still want to take part in the experiment and undergo screening procedures.

Cancer related medical history, blood and anthropometric measurements (BMI, hip circumference and lipid profile) performed by a registered health professional. Participants are screened to select those that meet the trial criteria. Participants that meet the criteria then complete a 24-hour blood pressure measurement, they are then randomized into one of two groups (placebo and RK). The subjects are required to keep a diary of the number of daily and nightly hot flush events at specified time points through-out the 3 months study period. Kupperman index questionnaires may be used to assess the intensity of the symptoms and sent out at weeks 0, 6 and 12 (containing the return envelope and postage stamp), and returned by the subject at the end of the week.

Participants contacted weekly by the investigator (Associate Professor Phd. Per Bendix Jeppesen) or one of the other participants to hear the subjects on the trial process. Every 3rd week of meetings participants up at Vendsyssel Hospital to pick up dose of placebo or RK extract. There are a total of 3 obligatory visits, weeks 0, 6 and 12, taking fasted urine and blood samples for analysis (lipids, inflammatory markers and isoflavones). At weeks 0, 6 and 12 a 24-hour measurement of the sweating and skin temperature it taken. The participant must eat a standard evening meal the night before and breakfast. At the beginning and end is also performed a 24-hour blood pressure measurement. All procedures will be described in SOPs (standard operating procedure).

  Overview of the study processes and two points are shown in Table 1, Table 2 and table 3

| Measurements (weeks) | -2 | 0 | 3 | 6 | 9 | 12 |
| --- | --- | --- | --- | --- | --- | --- |
| Clinical check up | X | X |  |  |  | X |
| Questionnaires |  | X | X | X | X | X |
| BMI | X | X |  |  |  | X |
| DEXA |  | X |  |  |  | X |
| Blood and urine metabolomics | X |  |  | X |  | X |
| Blood pressure and plasma sampling (lipids og inflammatory markers) | X |  |  | X |  | X |
| 24 hour blood pressure measurments |  | X |  |  |  | X |
| 24 hour ambulatory skin conductance |  | X |  | X |  | X |
| Blood sampling for compliance | X |  |  | X |  | X |
| Briefing and orientation | X | X | X | X | X | X |

Table 1 *Treatment of the participants and general measurement parameters*

| Blood samples (weeks) | -2 | 0 | 3 | 6 | 9 | 12 |
| --- | --- | --- | --- | --- | --- | --- |
| Lipid Profile (tryglicerides, LDL:HDL, FFA og Total cholesterol , ApoLp-a) | X |  |  | X |  | X |
| Isoflavone Compliance (Genstien, Diadzien, Formononetin, Bichanin A) | X |  |  | X |  | X |
| Cytokines (NF-κB ↓, IL6, TNFα, NOS and Cyc O 2: ↑IL 10) | X |  |  | X |  | X |
| Follicle Stimulating Hormone (FSH) and estrodiol | X |  |  | X |  | X |

Table 2 *Blood test parameters*

| Urine Samples (Weeks) | -2 | 0 | 3 | 6 | 9 | 12 |
| --- | --- | --- | --- | --- | --- | --- |
| Metabolomics  HPLC/MS | X |  |  | X |  | X |

Table 3 *Metabolomics of urine samples*

# Participants

A minimum of 50 women aged 40-65 years are required to complete the study based on our power calculation in the spring 2012. Participants must keep their physical activity, smoking and alcohol habits stable throughout the entire test. Use of blood pressure - and lipid-lowering drugs can be continued during the trial.

**Inclusion Criteria**:

Inclusion criteria:

• Age 40-65 years, only women.

• Do daily hot flashes

• Body Mass Index (BMI) between 20-40

• Have irregular bleeding

• FSH levels between 30-50 MUI / mL

**Exclusion Criteria**

# • Simultaneous participation in other clinical trials within the last 3 months

# • Deficient bleeding for more than 6 months

# • Excessive cardiovascular-, psychiatric-, neurological-, and / or kidney disease.

# • Alcohol or substance abuse and acute illness.

# • Blood pressure> 160/110

# • Pregnant and lactating women

# A participant excluded from the study if they repeatedly fail to follow the study guidelines.

# Recruitment of participants

# Participants are recruited by doctors via the outpatient clinic at Vendsyssel Hospital (contacting the outpatient clinic in connection with the attendance or any other reason), practitioners in the field and through ads in local magazines as Nordjyske, Vendelbo Posten (ad enclosed) and leaflets, then potential participants refer to the principle investigator Per Bendix Jeppesen. Participants are informed and briefed about the project by the research team over the phone. If the participant is still interested they are forwarded a written participant information brochure and "research participants' rights in a biomedical research" document published by the Central Research Ethics Committee. After the participants have had the opportunity to read the written information the participants are invited to an oral interview where project leader will be able to answer any questions, and later obtain consent. Participants are given at least one week to think from first oral information for obtaining consent. Participants have the opportunity to bring a a friend or family member for support. The subjects are informed that participation is voluntary and that they can at any time withdraw from the study without the influence on their treatment or control at the hospital. Participants are insured through the hospital's Patient Insurance.

# Handling of participants data and data protection

# Full details of the study participants are protected under the Act on Processing of Personal Data and Health Act (GCP regulations). All samples (blood and urine) will be coded (participant ID), so only the investigator / -centret can connect results to the individual participant after study completion. The results are confidential and shall not be disclosed to outsiders. The study reported to the Data Protection Agency.

# Publication

# Trial results, both positive and negative results will be included in scientific articles that will be published in scientific journals.

# Financing the study

# The study, conducted at the Center for Clinical Research, Vendsyssel Hospital, under the administration of Endocrinology Department. MEA (Aarhus University Hospital) funded by Agro Tech ltd.

# The initiative for the experiment is Associate Professor, PhD, Per Bendix Jeppesen, from the Endocrinology Department. MEA (Aarhus University Hospital).

# None of the participants in the study have financial interests linked to private companies or foundations involved in the study. The project complies with the Act on processing of personal data and the project notified to the DPA.

# There will be no fee for the subjects of participation. Be repaid for travel and transportation expenses and lost wages for government rates in the investigations at the hospital.

# Risks in participating in the study

Participants are requested to report all side effects that may be related to their treatment.

Blood sampling is associated with a small risk of perforation of the vein and a subsequent small, superficial hematoma. There is also a minimal risk of infection at the injection site.

There is a small radiation risk by DEXA scan, which is otherwise without discomfort. DEXA scanning constitutes a radiation dose from .02 to 0.05 mSv. examination. By comparison, the annual background radiation around 3 mSv.

# Ethics

# The risks and disadvantages that participation in the study may pose to the participants are discussed in the previous section. Participation in the study will have the following advantages: a thorough clinical and biochemical check-up and determination of bone density. At the end of the study all participants will be informed about their results. If blood tests show anything abnormal, the patient will be advised to contact their GP or will be referred to the relevant department at the hospital.

# The study will provide valuable new knowledge and documentation of red clover extract can alleviate women suffering from menopause-related symptoms. This knowledge can thus be a useful tool in the treatment of menopause.

# Overall, the study is estimated to be associated with minimal risks to participants and it will provide the participants with relevant information about their own health, and it will provide valuable biomedical knowledge about red clover extract as a new dietary supplement.

#

# References

1) J. Moilanen, A.-M. Aalto, E. Hemminki, A.R. Aro, J. Raitanen and R. Luoto (2010) Prevalence of menopause symptoms and their association with lifestyle among Finnish middle-aged women Maturitas: 67: 368–374

2) S. Palacios, V. W. Henderson, N. Siseles, D. Tan and P. Villaseca (2010) Age of menopause and impact of climacteric symptoms by geographical region. CLIMACTERIC 13: 419–428

3) Peter Chedraui, Glenda San Miguel, Luis Hidalgo, Nancu Morocho & Susana Ross (2008) Effect of Trifolium pratense-derived isoflavones on lipid profile of postmenopausal women with increased body mass index. Gynocological Endocrinology: 24 (11): 620-624

4) Stacy E. geller and Laura Studee (2006) Soy and red clover for midlife and aging. Climeractic: 9 (4): 245- 263

5) Joanna Thompson Coom, Max H. Pittler, Edward Ernst (2007) Trifolium Pratense isoflavones in the treatment of menopausal hot flushes: A systematic review and meta-analysis. Phytomedicine 14, 153-159

6) [Geller SE](http://www.ncbi.nlm.nih.gov/pubmed?term=%22Geller%20SE%22%5BAuthor%5D), [Shulman LP](http://www.ncbi.nlm.nih.gov/pubmed?term=%22Shulman%20LP%22%5BAuthor%5D), [van Breemen RB](http://www.ncbi.nlm.nih.gov/pubmed?term=%22van%20Breemen%20RB%22%5BAuthor%5D), [Banuvar S](http://www.ncbi.nlm.nih.gov/pubmed?term=%22Banuvar%20S%22%5BAuthor%5D), [Zhou Y](http://www.ncbi.nlm.nih.gov/pubmed?term=%22Zhou%20Y%22%5BAuthor%5D), [Epstein G](http://www.ncbi.nlm.nih.gov/pubmed?term=%22Epstein%20G%22%5BAuthor%5D), [Hedayat S](http://www.ncbi.nlm.nih.gov/pubmed?term=%22Hedayat%20S%22%5BAuthor%5D), [Nikolic D](http://www.ncbi.nlm.nih.gov/pubmed?term=%22Nikolic%20D%22%5BAuthor%5D), [Krause EC](http://www.ncbi.nlm.nih.gov/pubmed?term=%22Krause%20EC%22%5BAuthor%5D), [Piersen CE](http://www.ncbi.nlm.nih.gov/pubmed?term=%22Piersen%20CE%22%5BAuthor%5D), [Bolton JL](http://www.ncbi.nlm.nih.gov/pubmed?term=%22Bolton%20JL%22%5BAuthor%5D), [Pauli GF](http://www.ncbi.nlm.nih.gov/pubmed?term=%22Pauli%20GF%22%5BAuthor%5D) and [Farnsworth NR](http://www.ncbi.nlm.nih.gov/pubmed?term=%22Farnsworth%20NR%22%5BAuthor%5D). (2009) Safety and Efficacy of Black Cohosh and Red Clover for the Management of Vasomotor Symptoms: A Randomised Control Trial. [Menopause.](http://www.ncbi.nlm.nih.gov/pubmed/19609225) 16(6):1156-66.

7) V. Beck, U. Rohr, A. Jungbaur (2005) Phytoestrogens derived from red clover: An alternative to estrogen therapy? Journal of steroid biochemistry and molecular biology 94: 499 – 518

8) Marjus Lipovic, Peter Chedraui, Christine Gruenhut, Anca Gocan, Christine Kurz, Benedikt Neuber and Martin Imhof (2011) The effect of red clover isoflavones supplementation over vasomotor and menopausal symptoms in postmenopausal women. Gynocological Endocrinology 1-5

9) Heather G. Miller, MFS,PhD and Maria Li, MBA PhD (2004) Measuring Hot Flashes: Summary of a National institutes of Health Workshop. Mayo Clinic Proceedings 79: 777-781

10) J.G. Webster, D. E. Bahr, M. C. Shults, D. G. Grady and J. Macer (2004) A miniature sternal skin-attached hot flash recorder. National Centre for complementary and Alternative medicine, Bahr Management Inc.

11) Nelson HD, Haney E, Humphrey L, et al. (2005) Management of Menopause-Related Symptoms. Rockville (MD): Agency for Healthcare Research and Quality (US); (Evidence Reports/Technology Assessments, No. 120.)

12) Edwin D. Lephart, Kenneth D. R. Setchell, Robert J. Handa, and Trent D. Lund (2004) Behavioral Effects of Endocrine-disrupting Substances: Phytoestrogens. ILAR Journal 45 (4): 443-454
